# Inclusion of Plasma Lipid Species Improves Classification of Individuals at Risk of Type 2 Diabetes

**DOI:** 10.1371/journal.pone.0076577

**Published:** 2013-10-08

**Authors:** Gerard Wong, Christopher K. Barlow, Jacquelyn M. Weir, Jeremy B. M. Jowett, Dianna J. Magliano, Paul Zimmet, Jonathan Shaw, Peter J. Meikle

**Affiliations:** Baker IDI Heart and Diabetes Institute, Melbourne, Australia; University of Texas Health Science Center at San Antonio, United States of America

## Abstract

**Background:**

A significant proportion of individuals with diabetes or impaired glucose tolerance have fasting plasma glucose less than 6.1 mmol/L and so are not identified with fasting plasma glucose measurements. In this study, we sought to evaluate the utility of plasma lipids to improve on fasting plasma glucose and other standard risk factors for the identification of type 2 diabetes or those at increased risk (impaired glucose tolerance).

**Methods and Findings:**

Our diabetes risk classification model was trained and cross-validated on a cohort 76 individuals with undiagnosed diabetes or impaired glucose tolerance and 170 gender and body mass index matched individuals with normal glucose tolerance, all with fasting plasma glucose less than 6.1 mmol/L. The inclusion of 21 individual plasma lipid species to triglycerides and HbA1c as predictors in the diabetes risk classification model resulted in a statistically significant gain in area under the receiver operator characteristic curve of 0.049 (p<0.001) and a net reclassification improvement of 10.5% (p<0.001). The gain in area under the curve and net reclassification improvement were subsequently validated on a separate cohort of 485 subjects.

**Conclusions:**

Plasma lipid species can improve the performance of classification models based on standard lipid and non-lipid risk factors.

## Introduction

Global prevalence of diabetes mellitus is rising as a result of population ageing, changes in diet, reduced physical activity and rising obesity prevalence [1], [2]. In 2010, an estimated 285 million people worldwide had diabetes mellitus, 90% of whom had type 2 diabetes mellitus [1]. This figure is projected to rise to 439 million by 2030 [3].

In Australia, Magliano *et al.* [4] conservatively estimate at least 2 million adults with type 2 diabetes by 2025, and close to 3.5 million by 2033 [5]. This presents a burgeoning burden on health service resources.

Early intervention through lifestyle changes have been reported to have a positive effect on reducing the progression of those at risk of type 2 diabetes [6]. Early intervention through pharmacological treatment of impaired glucose tolerance (IGT) individuals at high risk of type 2 diabetes with oral antidiabetic agents that improve insulin sensitivity and preserve β-cell function have also been shown to reduce the progression of IGT to type 2 diabetes by 50–70% [7].

Type 2 diabetes risk prediction models can assist the decision-making process regarding the clinical management of a patient in terms of identifying a need for early intervention. The prediction models are typically multivariate, combining several disease risk factors to quantify a patient’s risk disposition to an outcome over a specified span of time in the future. On the basis of the risk score computed by the model, healthcare interventions or lifestyle changes can be recommended and targeted towards those at an increased risk of developing a disease or those likely to have an adverse clinical outcome such as patients with established clinical atherosclerosis [8], [9]. Noble *et al.* [10] performed a comprehensive review of 94 type 2 diabetes risk prediction models. Their findings summarised the most common components of type 2 diabetes risk prediction models to include non-lipid risk factors such as age, a measure of obesity such as body mass index (BMI), waist circumference or waist-to-hip ratio, gender, family history of diabetes, a measure of blood pressure such as systolic blood pressure, diastolic blood pressure or hypertension and fasting plasma glucose (FPG), two-hour postprandial plasma glucose or glycated haemoglobin (HbA1c). The common lipid risk factors incorporated into type 2 diabetes risk prediction models are high density lipoprotein cholesterol (HDL-C), low density lipoprotein cholesterol (LDL-C) and triglycerides. However, recent studies have suggested that specific lipid species may be useful markers of diabetes. For example, Haus *et al*. [11] showed that certain ceramide subspecies are elevated in obese subjects with type 2 diabetes and correlate with the severity of insulin resistance. While Boon *et al*. [12] showed that ceramides contained in LDL are elevated in type 2 diabetes and promote inflammation and skeletal muscle insulin resistance.

The Australian Diabetes, Obesity and Lifestyle (AusDiab) study is the largest Australian longitudinal population-based study examining the natural history of diabetes, pre-diabetes (combined impaired fasting glucose (IFG) and IGT), heart disease and kidney disease. The baseline study conducted in 1999–2000 provided benchmark national data on the prevalence of diabetes, obesity, hypertension and chronic kidney disease in Australia. The baseline study preceded two follow up studies spaced approximately five and seven years apart where similar extensive collection of data was performed [13], [14]. The classification of the subjects was determined by a fasting plasma glucose test and an oral glucose tolerance test. The thresholds for the fasting plasma glucose and 2 h post load glucose used to determine type 2 diabetes and pre-diabetes status were consistent with the recommended thresholds set by the International Diabetes Federation. At the five year follow-up of 6537 subjects, 225 had developed type 2 diabetes. This represented 15.8% of those who had IGT at baseline, 11.4% of those with impaired fasting glucose (IFG) and 1.2% of the normal glucose tolerance (NGT) group. These progression percentages of pre-diabetes (IFG+IGT) to type 2 diabetes highlight the increased risk in this group and the potential advantage, from a diabetes prevention point of view, to identify these individuals for the purpose of early intervention.

Individuals with FPG >7.0 mmol/L are diagnosed as having type 2 diabetes and individuals with FPG of between 6.1 mmol/L and 7.0 mmol/L (IFG) are usually followed-up with a subsequent oral glucose tolerance test which will confirm these individuals to having IGT, IFG or type 2 diabetes. However, a significant proportion of individuals with type 2 diabetes or IGT have FPG below 6.1 mmol/L. This subgroup in the population are often neglected and not followed-up on post type 2 diabetes screening. In this study, we set out to identify undiagnosed type 2 diabetes and IGT in individuals with an FPG <6.1 mmol/L with the perspective that these individuals can then proceed to receive the required treatment or early intervention. In the AusDiab study, individuals with an FPG <6.1 mmol/L account for approximately 35.3% of all individuals with either prevalent undiagnosed type 2 diabetes or IGT.

While standard plasma lipids (LDL-C, HDL-C and triglycerides) are recognised risk factors for type 2 diabetes [10], recent studies using lipidomic approaches have demonstrated that the individual lipid species present in lipoproteins show differential association with disease status and so may provide additional information relating to disease pathogenesis and disease risk [15]–[18]. In this study we have applied lipidomics to assess the utility of individual plasma lipid species in improving on standard lipid and non-lipid risk factors in the task of identifying undiagnosed type 2 diabetes and IGT in individuals with an FPG <6.1 mmol/L.

## Methods

### Ethics Statement

All participants in the AusDiab study provided informed written consent. This study was approved by the Ethics Committee of the Alfred Hospital (Project No: 104/10).

### Study Design

The initial cross-sectional substudy which this work is based on was designed to identify associations of individual plasma lipid species with type 2 diabetes and for this purpose 351 individuals were selected. 117 individuals had undiagnosed type 2 diabetes (cases). Controls were frequency selected to match the cases in terms of age, sex and BMI. The controls consist of 170 NGT, 45 IGT and 19 IFG.

**Figure 1:**
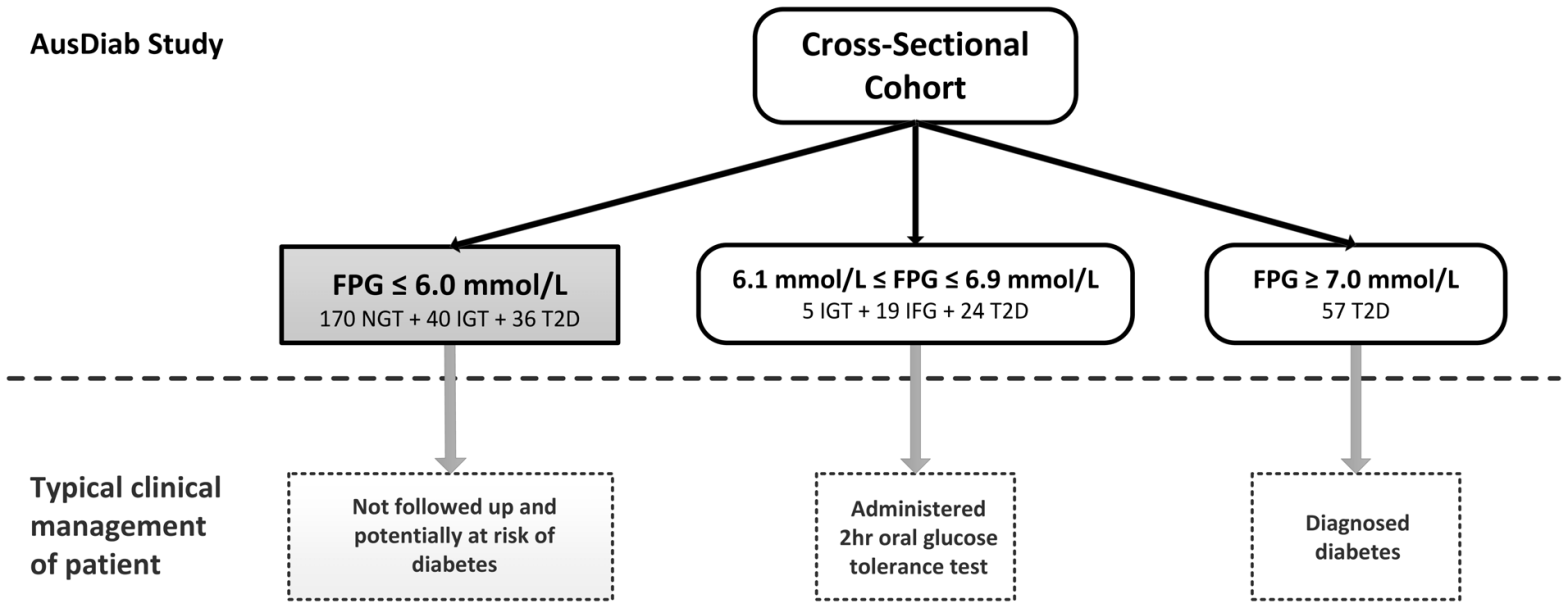
Distribution of patients in the AusDiab cross-sectional cohort and typical clinical management of patients in the various fasting plasma glucose categories. The top half of the figure describes the distribution of patients in the AusDiab cross-sectional cohort by FPG ranges while the lower half of the figure describes the typical clinical management of patients in the various FPG categories. Typical clinical management is distinct to patient management in the AusDiab study. In a typical clinical context, subjects with FPG ≥7.0 mmol/L are diagnosed immediately as having diabetes, subjects with an FPG between 6.1 mmol/L and 6.9 mmol/L are referred for a 2 hr oral glucose tolerance test to confirm their diabetes status (type 2 diabetes, IFG or IGT) and subjects with an FPG ≤6.0 mmol/L are not typically followed up. The subjects in the latter group may have diabetes or be at risk of diabetes.

In this analysis, we have utilised a subset of the 351 individuals described above, focusing on individuals with a FPG measurement of less than 6.1 mmol/L for the purpose of training our classification model to evaluate the utility of plasma lipid species in type 2 diabetes risk classification. This subset consisted of 246 subjects of whom 36 had undiagnosed type 2 diabetes, 40 had undiagnosed IGT and 170 had NGT. Figure 1 provides a detailed breakdown of the subject numbers for each FPG category. The original matching criteria do not apply in this selected subset. For convenience, we will label these subjects *the initial subset*.

### Validation Cohort

To validate the utility of including individual plasma lipids species in type 2 diabetes risk classification, a separate cohort consisting of 485 subjects with FPG <6.1 mmol/L were selected randomly from the AusDiab study where none of the selected 485 subjects were part of the initial subset. The validation set consisted of 101 subjects with IGT and 384 subjects with NGT which is representative of the relative class proportions in the Australian population for the given sample size.

### Sample Preparation and Lipid Extraction

The order of the plasma samples was randomised prior to lipid extraction and analysis for each cohort. Quality control plasma samples were included at a ratio of 1 quality control plasma sample to 11 subject plasma samples. Total lipid extraction from a 10 µL aliquot of plasma was performed by single phase chloroform: methanol (2∶1) extraction [19].

### High Performance Liquid Chromatography-mass Spectrometry Analysis

Lipid analysis was performed by liquid chromatography, electrospray ionisation-tandem mass spectrometry using an Agilent 1200 liquid chromatography system combined with an Applied Biosystems API 4000 Q/TRAP mass spectrometer with a turbo-ion spray source (350°C) and Analyst 1.5 data system [19]. We have previously reported the use of precursor ion, neutral loss scans and multiple reaction monitoring (MRM) scans to identify and measure the predominant lipid species of the following lipid classes: dihydroceramide (dhCer), ceramide (Cer), monohexosylceramide (MHC), dihexosylceramide (DHC), trihexosylcermide (THC), G_M3_ ganglioside (GM3), sphingomyelin (SM), phosphatidylcholine (PC), alkylphosphatidylcholine (PC(O)), alkenylphosphatidylcholine (plasmalogen, PC(P)), lysophosphatidylcholine (LPC), lysoalkylphosphatidylcholine (lysoplatelet activating factor, LPC(O)), phosphatidylethanolamine (PE), phosphatidylinositol (PI), phosphatidylserine (PS), phosphatidylglycerol (PG), cholesterol ester (CE), free cholesterol (COH), diacylglycerol (DG) and triaclyglycerol (TG) [19]–[21]. The abbreviations shown above are only used when referring to individual lipid species as in LPC 22∶6 which define a lysophosphatidylcholine with a fatty acid containing 22 carbons and six double bonds. For a number of the lipids which contain two fatty acid chains the MRM based measurements here do not directly determine the constituent fatty acids but rather the sum of the number of carbons and the sum of the number of double bonds across both fatty acids. Accordingly, we denote these species as the combined length and number of double bonds, e.g. PC 36∶4.

A total of 71 diacylglycerol and triacylglycerol species and 216 other lipid species were analysed in two separate experiments. Relative lipid amounts were calculated by relating the peak area of each species to the peak area of the corresponding stable isotope or non-physiological internal standard [19].

### Statistical Analysis

To train a classification model that best stratifies type 2 diabetes and IGT from NGT, we applied approaches in machine learning and utilised MATLAB 2012a and the libraries of LIBSVM 3.14 [22] to implement the multivariate classification modelling experiments. The classification models were based on C-support vector classification [23], [24] with a polynomial kernel, and were trained and tested within a 3-fold stratified cross validation framework on 246 subjects with FPG <6.1 mmol/L from the initial subset. The following features (predictors) were available for inclusion in the models: sex, age, systolic blood pressure, waist, total cholesterol, HDL-C, triglycerides and *HbA1c* and 287 individual lipid species. All lipid species measurements were log-transformed and missing lipid species measurements were imputed with a log-transformed value of zero. A missing lipid species measurement is indicative that the specific lipid species was below the limit of detection in the sample.

In 3-fold cross validation, the dataset is randomly divided into thirds. As we applied stratified 3-fold cross validation, every third maintains an equal class proportion of the subjects, i.e., they had the approximately equal numbers of type 2 diabetes, IGT and NGT. The classification model was trained on two thirds of the dataset (known as the training set of samples) and the trained classification model was subsequently tested on the remaining third (known as the test set of samples). The order of feature inclusion for training the classification model was based on the area under the receiver operator characteristic curve (AUC) achieved by each feature as assessed on the training set of samples. The feature with the highest AUC was first to be included in the model. With each new feature included in the model, the performance of the classification model in terms of specificity, sensitivity and AUC was recomputed.

After the completion of the first iteration, the thirds of samples were permuted and the classifier retrained on a different pairing of samples (i.e. thirds). Feature selection was repeated to determine the order of inclusion in the model and classifier performance was reassessed once again in this new iteration on a new set (third) of test samples. In 3-fold cross validation, there are three possible pairings of sets of training and test samples, after the 3 possible permutations were performed and assessed; the entire process was repeated 200 times with samples randomly assigned to a stratification segment each time. Repeating cross-validation multiple times helps to minimise sampling bias with respective to the allocation of test and training samples. The performance measures (specificity, sensitivity and AUC) over 600 iterations were computed and the mean and 95% confidence interval for each measure was subsequently calculated.

Modelling was performed with three different combinations of features,

- **risk factors:** sex, age, systolic blood pressure, waist, total cholesterol, HDL-C, triglycerides, *HbA1c*
- **lipid species:** 287 individual lipid species
- **combination of risk factors and lipid species:** sex, age, systolic blood pressure, waist, total cholesterol, HDL-C, triglycerides, *HbA1c* and 287 individual lipid species

For each model variant, the optimal operating point is regarded as the number of features required to attain maximum AUC.

For the validation of our results on an unseen set of subjects, each of the three model variants were trained on all 246 samples of the initial subset using on the *x* most frequently selected features where *x* corresponds to the optimal operating point for that model.

Each of the trained classification models were tested on the validation set of 485 subjects. As the lipid measures from the two cohorts were experimentally analysed at different time periods, we applied z-score transformation to the log-transformed lipid species measurements on both datasets separately to mitigate any potential experimental batch effects prior to training and testing the classification models.

## Results

In Table 1, we summarise the characteristics of the two groups of subjects in the AusDiab cross-sectional study used to train and test our type 2 diabetes risk classification models within a 3-fold cross validation framework. These two groups were collectively labelled *initial subset* earlier. The subjects in both groups have an FPG <6.1 mmol/L. The first group includes prevalent undiagnosed type 2 diabetes and IGT and the second group is NGT. The first group of subjects had higher levels of 2 hr post-load glucose and HbA1c as expected. They were also older, had higher mean systolic blood pressure, total cholesterol and triglycerides than the second group of subjects.

**Table 1:** Characteristics of the 246 AusDiab subjects in the initial subset (with FPG less than 6.1 mmol/L).

	Prevalent Undiagnosed Type 2 Diabetes & IGT (n = 76)^a^	NGT (n = 170)^a^	p-value^b^
Age (yrs)	71 (58–74)	60 (49–72)	4.8**×**10^−3^
Sex (male %)	43.4	47.6	0.69
Waist (cm)	92.1 (85.9–100.5)	90.3 (83.3–98.2)	0.24
Hip (cm)	103.4 (98.5–107.1)	103.3 (98.9–108.3)	0.72
Waist-to-hip ratio	0.90 (0.83–0.95)	0.86 (0.80–0.93)	0.13
FPG (mmol/L)	5.4 (5.1–5.7)	5.3 (5.1–5.6)	0.24
PLG (mmol/L)^c^	10.7 (8.9–11.9)	5.8 (4.8–6.6)	1.03**×**10^−34^
HbA1c (%)	5.3 (5.1–5.6)	5.1 (5.0–5.3)	4.04**×**10^−7^
Insulin (pmol/L)	91.0 (66.7–127.1)	83.7 (68.8–101.4)	0.13
HOMA-B (mmol/L)^d^	129.3 (107.0–163.7)	124.8 (107.6–148.7)	0.23
HOMA-S (mmol/L)^e^	49.3 (36.4–67.4)	55.3 (45.9–66.3)	0.08
BMI (kg/m^2^)	26.3 (24.4–29.5)	26.0 (23.6–27.9)	0.33
Systolic BP (mmHg)	144 (131–159)	133 (121–146)	1.6**×**10^−3^
Diastolic BP (mmHg)	74 (64–82)	70 (61–80)	0.24
Total Cholesterol (mmol/L)	6.30 (5.50–6.80)	5.70 (5.10–6.40)	1.4**×**10^−3^
HDL-C (mmol/L)	1.34 (1.13–1.65)	1.42 (1.20–1.67)	0.24
LDL-C (mmol/L)	3.83 (3.12–4.52)	3.67 (3.00–4.23)	0.18
Triglycerides (mmol/L)	1.90 (1.29–2.53)	1.20 (0.90–1.55)	7.0**×**10^−8^

a Represented as median (IQR).

b Mann Whitney U-test p-value, except for sex where the p-value is based on the chi-square test of proportions, p-values are corrected for multiple comparisons using the Benjamini-Hochberg approach.

c Post-load glucose.

d Homeostasis Model Assessment score for estimate of steady state beta cell function.

e Homeostasis Model Assessment score for estimate of insulin sensitivity.

**Figure 2:**
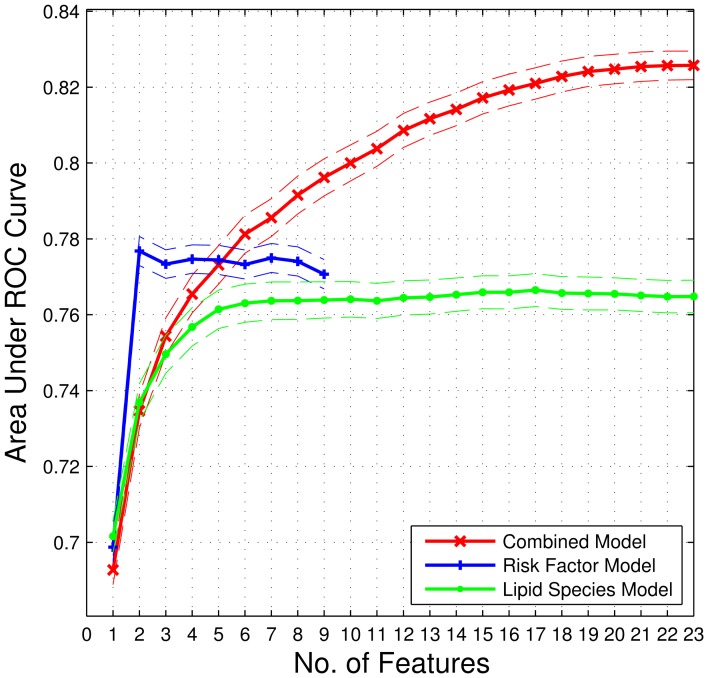
Plot of the area under the receiver operating characteristic curves (AUC) over an increasing number of features included in type 2 diabetes risk classification models. The combined model which includes sex, age, systolic blood pressure, waist, total cholesterol, HDL-C, triglycerides and *HbA1c* and 287 individual lipid species as features in the model attained a maximum AUC of 0.826 using 23 features the most frequent of which are listed in Table 4. Solid lines denote the mean AUC and broken lines represent the corresponding 95% confidence intervals plotted against the number of features incorporated into the model. The plot is truncated at 23 features.

Classification models were created using risk factors alone, lipid species alone or a combination of risk factors and lipid species. Figure 2 is an illustrative plot of how AUC changed as an increasing number of features were included in each model. Models created using risk factors alone (up to nine) showed an increase in the area under the ROC curve for only the first two risk factors (Figure 2). Table 2 shows that the two most frequently incorporated features in training the type 2 diabetes risk classification models based solely on risk factors were triglycerides and HbA1c, the rest of the risk factors were incorporated in a 2 risk factor type 2 diabetes risk classification model with a frequency of less than 2%.

**Table 2:** Most frequently incorporated features in a 2-feature risk factor only classification model.

Rank^a^	Feature	Frequency of Incorporation (%)
1	Triglycerides	99.0
2	HbA1c	98.8
3	Systolic Blood Pressure	1.50
4	Age	0.33

a No other risk factor features were included in a 2 risk factor model.

Table 3 describes the 17 most frequently incorporated lipid species features in training the type 2 diabetes risk classification model based solely on lipid species. Again, we focussed on the top 17 features in this context since the maximum AUC is achieved with 17 lipids (Figure 2). Within the top 17 features, 7 diacylglycerol species were incorporated in more than 97% of the models trained. Overall, only five classes of lipid species i.e., diacylglycerols, triaclyglycerols, cholesterol esters, phosphatidylethanolamines and dihydroceramides were reflected in the top 17 most frequently incorporated features in the type 2 diabetes risk classification model based solely on lipid species.

**Table 3:** Most frequently incorporated features in a 17-feature lipid species only classification model.

Rank^a^	Feature	Frequency of Incorporation (%)
1	DG 16∶0/22∶5	100
2	DG 16∶0/22∶6	100
3	DG 16∶1/18∶0	100
4	DG 16∶1/18∶1	100
5	DG 16∶0/16∶0	99.8
6	DG 18∶0/18∶1	99.5
7	DG 16∶0/18∶0	97.3
8	dhCer 18∶0	90.3
9	PE 40∶6	86.2
10	TG 14∶1/16∶1/18∶0	78.3
11	TG 16∶1/16∶1/16∶1	75.2
12	DG 18∶0/18∶2	69.8
13	CE 24∶1	57.0
14	DG 16∶0/20∶4	56.5
15	DG 14∶0/18∶1	48.0
16	DG 16∶0/20∶3	43.2
17	CE 22∶0	38.5

a For conciseness only the top 17 features in a 17 lipid species model are listed.

Table 4 describes frequently incorporated features in a type 2 diabetes risk classification model based on a combination of risk factors and lipid species. 23 features were required in this scenario to attain maximum AUC (Figure 2). Six diacylglycerol species and triglycerides were always incorporated in a 23 feature model consisting of both risk factors and lipid species with the rest of the features consisting of HbA1c, diacylglycerols, triaclyglycerols, cholesterol esters and 1 species each of phosphatidylethanolamine and dihydroceramide.

**Table 4:** Most frequently incorporated features in a 23-feature combined risk factor and lipid species classification model.

Rank^a^	Feature	Frequency of Incorporation (%)
1	DG 16∶0/16∶0	100
2	DG 16∶0/22∶5	100
3	DG 16∶0/22∶6	100
4	DG 16∶1/18∶0	100
5	DG 16∶1/18∶1	100
6	DG 18∶0/18∶1	100
7	Triglycerides	100
8	DG 16∶0/18∶0	99.3
9	HbA1c	94.8
10	dhCer 18∶0	94.7
11	PE 40∶6	93.7
12	TG 14∶1/16∶1/18∶0	93.0
13	TG 16∶1/16∶1/16∶1	88.8
14	DG 18∶0/18∶2	85.3
15	DG 16∶0/20∶4	73.5
16	DG 14∶0/18∶1	72.0
17	CE 24∶1	70.5
18	DG 16∶0/20∶3	65.0
19	DG 14∶0/16∶0	54.2
20	TG 14∶0/16∶0/18∶2	54.0
21	TG 16∶0/17∶0/18∶2	52.8
22	CE 22∶0	49.7
23	DG 14∶0/18∶2	48.2

a For conciseness, only the top 23 features in a 23 combined feature model are listed.

Table 5 is a detailed summary of the performance of type 2 diabetes risk classification models based solely on risk factors, lipid species and a combination of risk factors and lipid species. The optimal operating point specified in the table reflects the number of features used to attain the maximum AUC. The risk factor only model consisting of sex, age, systolic blood pressure, waist, total cholesterol, HDL-C, triglycerides and *HbA1c* achieved an AUC of 0.777 at an optimal operating point of two features. *T*he lipid species only model achieved an AUC of 0.765 at an optimal operating point of 17 features where the majority of lipid species in this model were diacylglycerols and triaclyglycerols. The combined lipid and risk factor model consisting of the entire complement of features described in the two previous models achieved an AUC of 0.826 at an optimal operating point of 23 features. The two non-lipid species features included in the top 23 most frequently incorporated features were HbA1c and triglycerides as expected. The combined model had the highest sensitivity (57.6%) among the three models and comparable specificity (91.3%) to the rest of the models.

**Table 5:** Summary of classification model performance.

Model	A^a^	B^b^	A+B^c^
Operating Point^d^	2	17	23
Area under ROC curve	0.777 (0.773, 0.781)	0.765 (0.760, 0.769)	0.826 (0.822, 0.83)
Gain in AUC^e^			0.049
Sensitivity (%)	44.0 (43.1, 44.9)	46.5 (45.8, 47.3)	57.6 (56.9, 58.3)
Specificity (%)	94.4 (94.1, 94.8)	90.3 (89.9, 90.6)	91.3 (91, 91.6)
NRI^f^ (%)			10.5
Classification Accuracy^g^ (%)			
Type 2 diabetes	45.7 (44.7, 47.2)	52 (50.8, 52.9)	66.2 (65.3, 67.4)
IGT	42.5 (41.3, 43.3)	41.6 (40.5, 42.5)	49.9 (48.9, 50.9)
NGT	94.4 (94.1, 94.8)	90.3 (89.9, 90.6)	91.3 (91, 91.6)

a Risk factor model.

b Lipid species model.

c Combined risk factor and lipid species model.

d Number of features required to attain maximum AUC.

e Difference in AUC between model A+B and model A.

f Net reclassification improvement between model A+B and model A.

g Correct classification of type 2 diabetes and IGT into the group requiring treatment or intervention and NGT into the group not requiring treatment or intervention expressed as a percentage of the total number of subjects in the type 2 diabetes, IGT and NGT groups individually.

The difference in mean AUC of 0.049 between the risk factor model and the combination risk factor and lipid species model was statistically significant (p<0.001) and this translates into a statistically significant net reclassification improvement (NRI) of 10.5% (p<0.001). Using the combined risk factor and lipid species model, a mean of 66.2% of undiagnosed type 2 diabetes and 49.9% of IGT were classified as requiring treatment or intervention while a mean of 91.3% of NGT subjects were classified as not requiring treatment or intervention.

In Table 6, we summarise the performance of each of the three type 2 diabetes risk classification model variants tested on the unseen cohort of 485 subjects. The gain in AUC and net reclassification improvement were confirmed on a randomly selected group of 485 AusDiab subjects with fasting glucose measurement <6.1 mmol/L (excluding all subjects from the initial cohort), where the combined risk factor and lipid species risk classification model achieved a gain in AUC of 0.044 and an NRI of 18.7% over the risk factor only model.

**Table 6:** Performance of different classification models on the validation cohort.

Model	α^a^	β^b^	α+β^c^
Operating Point	2	17	23
Area under ROC curve	0.684	0.668	0.729
Gain in AUC^d^			0.044
Sensitivity	32.7	34.7	53.4
Specificity	86.2	83.1	83.8
NRI^e^			18.3%

a Risk factor model trained on the AusDiab cross-sectional study and tested on validation dataset.

b Lipid species model trained on the AusDiab cross-sectional study and tested on the validation dataset.

c Combined risk factor and lipid species model trained on the AusDiab cross-sectional study and tested on the validation dataset.

d Difference in AUC between model α+β and model α.

e Net reclassification improvement between model α+β and model α.

## Discussion

Plasma lipid profiling of two separate cohorts of the AusDiab study provided us with the ability to evaluate the capacity of plasma lipids to improve on traditional risk factors for the identification of prevalent undiagnosed type 2 diabetes and pre-diabetes. The results demonstrate that the addition of plasma lipid species to triglycerides and HbA1c significantly improves the classification of individuals at risk of type 2 diabetes. The gain in AUC of 0.044 and an NRI of 18.7% over the risk factor only model in the validation set of subjects confirms that the inclusion of plasma lipid species to standard lipid and non-lipid risk factors, specifically, HbA1c and triglycerides, improves type 2 diabetes risk classification performance.

In the initial subset, our objective was to identify individuals with prevalent undiagnosed type 2 diabetes or IGT from individuals with NGT. The stratification of subjects based on FPG in the cross-sectional study is shown in Figure 1. Individuals with FPG ≥6.1 mmol/L are identified as having type 2 diabetes or IFG. Subsequent development of multivariate classification models to identify IGT and prevalent undiagnosed type 2 diabetes from NGT in individuals with FPG <6.1 mmol/L identified triglycerides and HbA1c as the principal drivers of risk stratification in a cohort which was generally well-matched in characteristics between the NGT and non-NGT groups as described in Table 1.

When plasma lipid species were included as predictors in the model, classification performance improved and we observed diacylglycerol to be the major lipid class most frequently incorporated, representing seven of the top eight ranked lipid species, although dihydroceramide, phosphatidylethanolamine, triacylglycerol and cholesterol ester species were also frequently incorporated. The significance of this result lies in the fact that these diacylglycerol species are able to contribute independently to the stratification of the groups beyond total triglycerides, implying that they do not simply reflect the elevated triglyceridemia present in the high risk individuals but rather reflect a separate biological process. Stahlman *et al*. [25] recently reported on the composition of VLDL and LDL from dyslipidemic women with type 2 diabetes; diglycerides were significantly elevated whereas triglycerides showed a non-significant increase. They also observed an enrichment of palmitate (16-carbon saturated fatty acid) in both diacylglycerol and triacylglycerol species which is also in agreement with our observations of the most frequently incorporated species (DG 16∶0/16∶0, DG 16∶0/22∶5 and DG 16∶0/22∶6, Table 4). They propose that the inability to observe a significant association between triacylglycerol species and type 2 diabetes may relate to the rapid metabolism of the large triacylglycerol rich VLDL particles. While diacylglycerol has been implicated in the aetiology of insulin resistance in both muscle and liver by its activation of protein kinase C-ε (PKCε) [26]–[28], it is, at present, unclear if this can be mediated by the diacylglycerol rich lipoproteins identified in these studies.

Some type 2 diabetes risk prediction models are heavily weighted on obesity. However, obesity is not always associated with deleterious changes to the lipidome associated with increased risk of diseases such as type 2 diabetes and coronary heart disease. For instance, a recent study by Ortega *et al*. [29], found that 46 percent of all obese adults in their study were metabolically healthy, i.e. they did not have high blood pressure, high blood cholesterol, or a high blood sugar levels and had a 38 percent lower risk of mortality from any cause compared to metabolically unhealthy obese people. Being obese in this instance did not predispose these individuals to an increased risk of disease over their non-obese counterparts as the physiological characteristics of these individuals were not translated into deleterious lipidomic profiles. This opinion is consistent with an earlier commentary [30] which identified the metabolically benign obese to have favourable metabolic profiles, high insulin sensitivity, no hypertension, normal lipid, inflammation and hormonal profiles (low triglycerides and C-reactive protein concentrations and high HDL-C and adiponectin concentrations) making them almost indistinguishable from young lean individuals in this regard. Stefan *et al*. [31] also described the potentially protective metabolic profile of these individuals to consist of lower visceral, liver and muscle fat content compared to insulin-resistant obese people. Additionally, they had a better ability to trap free fatty acids in adipose tissue and had lower carotid intima-media thickness which also suggests a favourable cardiovascular profile.

With our ability to measure 287 individual lipid species in plasma through the use of liquid chromatography electrospray ionisation tandem mass spectrometry, we have obtained a more detailed view of specific lipid species that are tightly coupled to mechanisms associated with the pathogenesis of type 2 diabetes. As the groups were matched for BMI during selection, there was no resultant significant difference in waist circumference (a surrogate measure for visceral obesity) between the groups and thus waist circumference does not improve the discrimination power of the model. The matching of waist circumference however enhances the efficiency of our analysis in identifying lipid species that may contribute additionally and independently to waist circumference in risk stratification for type 2 diabetes.

Although the groups in this study were matched for age, sex and BMI, plasma lipid species were still able to significantly improve on type 2 diabetes risk classification performance when added to triglycerides and HbA1c as predictors in the diabetes risk classification model. This suggests that plasma lipid species are able to capture some of the more subtle signatures of diabetes risk beyond risk factors such as BMI, sex, triglycerides and HbA1c. These results highlight the potential utility of plasma lipid species in the diagnosis of type 2 diabetes.

### Limitations of the Study

The current study is of cross-sectional design and of moderate size. To maximise the utilization of available samples and to minimise sampling bias, we have employed a 3-fold cross validation framework repeated 200 times to assess the performance of the trained models and to obtain corresponding confidence intervals for the various measures of performance. We have also validated the utility of lipid species in the context of type 2 diabetes risk classification on an unseen set of 485 subjects from the AusDiab study from which the gain in AUC and NRI from our cross-validated models were confirmed. This provides strong evidence that our results have not occurred by chance. We were however unable to obtain plasma samples for undiagnosed diabetes in the validation set of subjects, the inclusion of which we feel may further improve our validation performance results. A non-trivial extension of the current work to include the entire AusDiab study would provide us with the ability to perform a rigorous cost-benefit analysis to assess the feasibility of including plasma lipids species in a clinical diagnostic/screening model for the classification of type 2 diabetes risk in an actual population setting. Such larger validation studies will pave the way for the translation of this technology into clinical use for population-based risk screening of type 2 diabetes.
